# Pre-shot combinations and game-related statistics discriminating between winners and losers depending on the game location during the NBA COVID-19 season

**DOI:** 10.3389/fphys.2022.949445

**Published:** 2022-08-31

**Authors:** Alvaro Bustamante-Sánchez, Miguel-Angel Gomez-Ruano, Vicente Javier Clemente-Suárez, Sergio L. Jiménez-Sáiz

**Affiliations:** ^1^ Faculty of Sport Sciences, Universidad Europea de Madrid, Madrid, Spain; ^2^ Facultad de Ciencias de la Actividad Física y del Deporte, Universidad Politécnica de Madrid, Madrid, Spain; ^3^ Centre for Sport Studies, Fuenlabrada, Universidad Rey Juan Carlos, Madrid, Spain

**Keywords:** basketball, pick-and-roll, ball screen, game-related statistics, pre-shot combinations

## Abstract

Basketball in performance depends on numerous factors, where a stable trend was identified by winning teams with better performances in shooting effectiveness and rebounding. However, there is a need for a better understanding of pre-shot combinations that lead to these performance trends. This study aimed to analyze NBA teams’ game-related statistics, pre-shooting combinations, and pick-and-roll differences between winning and losing teams (considering the context: playing at home, away, or in a neutral court) during the COVID-19 season. A retrospective cross-sectional study on the 2019–2020 NBA season (906 games) was carried out. Game-related statistics were gathered from the private company InStat (https://basketball.instatscout.com/). The discriminant analysis and binary logistic regression models were run in order to discriminate the most important features of winning teams depending on the game location. The results showed that defensive rebounds and three-point shooting percentage remained the most important variables that best discriminated winners and losers independently of the game location context. The main results showed that winning teams had a better shooting percentage based on three-pointers, catch-and-shot actions, cuts, pick-and-roll efficacy, and uncontested shots based on a better collective behavior after a successful space creation dynamic through a tactical functional unit. At the same time, teams would need players with the ability to clear those possessions in which the opponents force to an isolation or a contested shot. From a practical application perspective, coaches should focus on composing a team with good shooters, skilled players in isolations, and a good game-time pick-and-roll strategy.

## Introduction

Basketball performance is dependent on several factors; hence, there exists a clear difficulty to predict and assess wins and defeats according to finite performance indicators (Gómez et al., 2017). However, due to its importance, much research has been previously conducted on this topic with the help of game-related statistics (Sampaio & Janeira, 2003; Evangelos et al., 2005; Gómez et al., 2008b; Ibáñez et al., 2008; Conte et al., 2017; Zhang et al., 2019). Undoubtedly, scoring points and thus having a good shooting effectiveness has a closed relationship with winning games (García et al., 2014; Gómez et al., 2008a). Traditionally, different studies have pointed out the importance of rebounding to recover the ball and then create fast-break or transition opportunities (Evangelos et al., 2005; Conte et al., 2017; Zhang et al., 2019). Particularly, defensive rebounding seems to be a key factor (Gómez et al., 2008a; Gómez et al., 2008b; Sampaio & Janeira, 2003) because of three main reasons: a better positioning, the opportunity to get back the ball, and the opportunity to avoid the other team to get an offensive rebound with more choices to score (closer to the basket and with no clear match-up) (Csátaljay et al., 2017). Other variables, like blocks, steals, or assists, seem to be related to a better performance; however, they were not reported as stable indicators helping to win games (Gómez et al., 2008a; Madarame, 2017; Çene, 2018). In this line, assists seem to be closely related to a better collective behavior and could not be counted without the occurrence of a scored basket (Gómez et al., 2008b). The importance of one-handed passes performed with the right hand has also been highlighted as a predictor of assists in winning teams (Gryko et al., 2020), and a better shooting performance, especially in two-point shots, has also been related to a better performance (Gryko et al., 2018).

Not only the game-related statistics but also the task-related performance indicators need to be accounted for (Gómez et al., 2017). The quantification of these factors can help to assess the quality of shots or the number of players involved in the last moments of ball possessions and control for the cooperation and opposition behaviors that happen before the action (Garganta, 2009; McGarry, 2009). Among these task-related performance indicators, ball screens are one of the most used tactical behaviors in professional basketball games, especially at the end of ball possessions (Gómez et al., 2015). The importance of this variable has consistently grown in the last 20 years (Remmert, 2003; Lamas et al., 2011; Gómez et al., 2013) and has been defined as a key factor to succeed in basketball with a consideration of functional units (Garganta, 2009). There are also other successful indicators for shooting, like the efficacy in uncontested and contested shots. Uncontested shots are usually preceded by a successful space creation dynamic through a tactical functional unit, while contested shots usually occur as a result of a successful space protection dynamic in the defensive phase (Lamas et al., 2015).

Game location is a contextual-related factor that could also affect the success during a game (Higgs & Stavness, 2021; Vaudreuil et al., 2021; Bustamante-Sánchez et al., 2022). Winning odds increase when the team has a home-court advantage because it may affect psychologically, physiologically, or has a direct impact on players’ performance, especially in rebounding actions (Leota et al., 2021). The 2019–2020 season provided a unique opportunity to perform a natural experiment including neutral-court games as a control group of games played without fans. The analysis of COVID-19 pandemic has been recently highly investigated in several team sports, but mainly in soccer (Lago-Peñas & Gómez-Ruano, 2021). The influence of no fans attending the matches clearly reflected the high impact that the crowd support has on teams’ and players’ performance (Tilp & Thaller, 2020), as some of the studies identified a reduction on the home advantage values (Correia-Oliveira & Andrade-Souza, 2021) and the changes in some key performance indicators when playing without fans (e.g., more yellow cards and fouls awarded to the local team in soccer). In particular, basketball research has only identified the home advantage trends without analyzing the key performance indicators related to each specific context during the COVID-19 pandemic (Alonso et al., 2022). Indeed, the NBA league can be studied from a specific analysis of three different contexts (home, away, and neutral courts) accounting for game-related statistics and key performance indicators (i.e., group-tactical behaviors) trying to identify if this neutral environment (no fan scenario) may reflect some fingerprints and specificities of team’s performance as was reflected in soccer.

Thus, this study aimed to analyze the teams’ performance differences (game-related statistics, pre-shooting combinations, and pick-and-roll performance) among winners and losers, considering the game locations (neutral court, home court, and away court), to better understand the performance indicators which best discriminate between the winning and losing teams. We hypothesized that winners would have better shooting percentages and rebounds; more transitions and catch-and-shot success; and more pick-and-roll and uncontested shots.

## Materials and methods

### Design

To analyze the performance indicators related to the game result and game location, a retrospective cross-sectional study on the 2019–2020 NBA season was carried out. Game data were collected from a commercially accessible provider (InStat, Moscow, Russia). According to previous studies, we selected the following performance indicators:

Game-related statistics: number of ball possessions, points per possession, points, field goals made, field goals attempted, field-goal percentage, two-point field goals made, two-point field goals attempted, two-point field goal percentage, three-point field goals made, three-point field goals attempted, three-point field-goal percentage, free throws made, free throws attempted, free-throw percentage, rebounds, offensive rebounds, defensive rebounds, assists, steals, turnovers, blocks, fouls, and fouls drawn.

Pre-shot combinations: transitions made, transitions attempted, percentage of offensive transitions, catch-and-shoot made, catch-and-shoot attempted, catch-and-shoot percentage, catch-and-drive made, catch-and-drive attempted, catch-and-drive percentage, post-up made, post-up attempted, post-up percentage, isolations made, isolations attempted, isolation percentage, hand-off made, hand-off attempted, hand-off percentage, cuts made, cuts attempted, cut percentage, drives made, drives attempted, and drive percentage.

Pick-and-roll (PnR), pick-and-pop (PnP), and defensive parameters: PnR handlers made, PnR handlers attempted, PnR handler percentage, PnR rollers made, PnR rollers attempted, PnR roller percentage, PnP made, PnP attempted, PnP percentage, uncontested field goals made, uncontested field goals attempted, uncontested field-goal percentage, contested field goals made, contested field goals attempted, and contested field-goal percentage.

Table 1 shows the action definitions that InStat provides.

**TABLE 1:** Action definitions by InStat.

Action	Definition
Possession	Ball possession by the player/team is a state when the player/team controls the ball. Possession lasts since throw-in/catching the ball till FG/losing the ball. Ball possessions are manually counted by InStat
Field goals attempted	Player’s action aimed at scoring the ball into the opponent’s basket. Shot percentage is the ratio of shots made to shots attempted
Two-PT field goals	Field goal attempt made inside of three-point line or when the player (his foot) touches it
Three-PT field goals	Field goal attempt in a basketball game made from beyond the three-point line
Free throws	Shot from a free-throw line which is awarded after a foul on the shooter by the opposing team or after going over the foul limit
Assist	Pass to a teammate that directly leads to a made field goal
Block	Defensive action when a defensive player legally deflects a field goal attempt from an offensive player by “covering” it with hand(s)
Foul	Breach of the rules. There are personal, technical, unsportsman-like, flagrant fouls, and team fouls. The game is stopped after the foul and followed by free-throw or an inbound situation
Foul drawn	A foul made by the opponents. The game is stopped after the foul and followed by free-throw or an inbound situation
Rebound	Retrieving the ball bouncing off the rim or backboard or after a missed shot. If the player from a defending team regains the ball, this is “defensive rebound.” If the ball is recovered by the offensive side, this is “offensive rebound”
Turnover	Action resulted in losing the ball by the team on offense to the opposing team
Steal	Action when the defending player causes turnover either by taking away the ball or stealing opponent’s pass
Contested/uncontested shot	If there is an opponent between the rim and a shooter, it is a contested shot. If there is no opponent, it is a uncontested shot
Transitions	Ball possessions which start at team’s backcourt and last more than 4 s and less than 8 s
Pick and roll	An offensive play in which a player sets a screen (pick) for a teammate handling the ball and then slips behind the defender (rolls). There are two types: pick-and-roll-handler—when a ball handler makes a shot attempt; pick-and-roll-roller—when a screener makes a shot
Isolation	An offensive play when a team gives the ball handler room to play one-on-one against his opponent
Catch and shoot	A play which was finished by a jumping shot at least 3 m from the rim by a player who controlled the ball less than 2 s or did not dribble
Post-up	A play when an offensive player receives the ball within 4.5 m with his back to the basket and making a shot attempt
Hand-off	A play when a ball handler is squeezed between his opponent and teammate and passes the ball to his teammate for a shot attempt
Cut	A play when a player attempting to shoot receives the ball while running toward the rim. It also includes screens, fast breaks, and situations when a player gets open at the rim
Catch and drive	A play which was finished by a shot at 3 m or less from the rim by a player who dribbled the ball after a pass
Drive	A play which was finished by a shot at 3 m or less from the rim by a player who dribbled

### Sample

A total of 906 games were analyzed during the 2019–20 NBA season. To analyze the performance indicators in the three different locations (playing at home *n* = 355, away *n* = 355, or in a neutral court *n* = 196), all the games played by the 22 teams who participated in the NBA (including the last part of the season) were included.

### Procedure

For data acquisition, InStat basketball reports (InStat, Moscow, Russia) were used. For each game, performance indicators were classified into two groups of analysis, depending on the game result: winners and losers. These groups were subsequently categorized in subgroups depending on the game location: home, away, or in a neutral court. To assess the validity and reliability of the data, a subsample of 10 games were randomly selected and were analyzed by two experienced coaches (kappa values >0.81).

The aim of this study was to investigate the technical and tactical actions of NBA teams in three different contexts (home, away, and neutral courts). Then, as the groups were different contexts, the observations were considered independent units of analysis. In particular, every single tactical action and group-tactical behavior that occur during a match configures a unique scenario of interactions of confronting teams, reflecting an unpredictable context where the players perform (Duarte et al., 2012). In addition, as the contextual-related factors were considered and the actions did not occur simultaneously, it allowed to use them as independent observations comparing those three contexts without affecting the units of analysis.

### Statistical analysis

Normality assumptions were checked using the Kolmogorov–Smirnov test. Homoscedasticity assumptions were checked using the Levene test. Descriptive statistics were presented as mean and standard deviation. A factorial two-way ANOVA test was used to compare the effect of the game result (win and lose), the effect of the game location (neutral-court, home-court, and away-court), and the interaction among the result and the game location. A Bonferroni *post hoc* test was used to analyze pairwise comparisons. The level of significance for all the comparisons was set at *p* < 0.05. The effect size was assessed by the eta-squared value (*η* ^*2*^) as specified in previous research (Fritz et al., 2012). A discriminant analysis was performed to identify the variables that best discriminate between the winning and losing teams. To assess the variables, we examined the structured coefficients greater than |0.30| (Ibáñez et al., 2008). Then, we used a binary logistic regression model, which is a nonlinear technique that estimates the coefficients that account for a change in the corresponding explanatory variable (Gómez et al., 2019). Their 95% confidence intervals (CIs) were also determined. The IBM SPSS statistical package version 21.0 for Windows (IBM Corp., Armonk, NY, United States) was used to analyze the data.

## Results

Table 2 shows the performance differences of game-related statistics (per possession) between winning and losing teams. Winning teams had better results in points, field goals made, field-goal percentage, two-point field goals made, three-point field goals made, three-point field-goal percentage, free-throw attempted, rebounds, defensive rebounds, assists, and steals. Neutral-court winners had better free-throw percentage and less turnovers. Home-court winners had more three-point field-goal attempts and blocks but less field-goal attempts and fouls. Away-court winners had better free-throw percentage and blocks but less fouls. The effect size for all performance indicators was small.

**TABLE:** 2Results of game-related statistics (per possession).

	Neutral				Home				Guest						
	Lost		Win		Lost		Win		Lost		Win		Game location and result		
	M	SD	M	SD	M	SD	M	SD	M	SD	M	SD	F	p	η^2^
Possessions	108.6	6.53	109.1	6.75	109.2	5.91	109.3	5.97	109.1	6.17	109.2	5.86	0.221	0.801	0.000
Points	0.98	0.09	1.08*	0.10	0.98	0.08	1.08*	0.09	0.97	0.96	1.06*	0.09	0.023	0.997	0.000
FG made	0.35	0.04	0.38*^,‡^	0.04	0.36	0.04	0.39*^,‡^	0.04	0.35	0.04	0.39*	0.04	0.203	0.817	0.000
FG missed	0.45^‡‡,‡‡‡^	0.05	0.41^‡‡,‡‡‡^	0.06	0.46^‡^	0.06	0.41*^,‡^	0.05	0.46^‡^	0.05	0.42^‡^	0.06	1.764	0.172	0.002
FG %	44.0	4.75	48.3*	5.23	43.8	5.11	48.9*	4.85	43.6	4.50	48.1*	5.18	0.973	0.378	0.001
Two-PT FG made	0.24^‡‡,‡‡‡^	0.04	0.25*^,‡‡,‡‡‡^	0.05	0.25‡	0.05	0.27*^,‡,‡‡‡^	0.04	0.25‡	0.04	0.26*^,‡,‡‡^	0.04	1.133	0.322	0.001
Two-PT FG missed	0.23^‡‡,‡‡‡^	0.05	0.21^‡‡,‡‡‡^	0.04	0.25^‡^	0.05	0.23^‡^	0.05	0.25^‡^	0.05	0.23^‡^	0.05	0.339	0.712	0.000
Two-PT FG %	51.4	6.77	54.9	7.28	50.9	7.72	55.1	6.56	50.4	6.45	54.2	7.19	0.307	0.735	0.000
Three-PT FG made	0.11^‡‡‡^	0.03	0.12*^,‡‡^	0.03	0.10	0.03	0.11*^,‡^	0.03	0.10^‡^	0.03	0.12*	0.03	0.986	0.373	0.001
Three-PT FG missed	0.22^‡‡‡^	0.05	0.20^‡‡,‡‡‡^	0.04	0.22	0.05	0.19*^,‡,‡‡‡^	0.04	0.21^‡^	0.05	0.19^‡,‡‡^	0.05	4.133	0.016	0.005
Three-PT FG %	33.6	8.03	38.8*	7.79	32.6	7.94	38.6*	8.28	32.6	7.74	38.6*	7.93	0.307	0.736	0.000
FT made	0.16^‡‡‡^	0.05	0.18^‡‡,‡‡‡^	0.05	0.15	0.05	0.17^‡^	0.05	0.15^‡^	0.05	0.16^‡^	0.05	0.340	0.712	0.000
FT missed	0.05^‡‡‡^	0.02	0.05*^,‡‡,‡‡‡^	0.03	0.05	0.02	0.05*^,‡^	0.05	0.05^‡^	0.02	0.05*^,‡^	0.02	0.055	0.946	0.000
FT %	77.9	8.62	79.9*^,‡‡^	9.45	76.5	9.21	77.8^‡^	9.77	76.3	11.1	78.4*	9.61	0.438	0.645	0.000
Rebounds	0.38	0.05	0.41*^,‡‡^	0.05	0.40^‡‡‡^	0.05	0.43*^,‡^	0.05	0.39^‡‡^	0.05	0.42*	0.05	0.481	0.618	0.001
Offensive rebounds	0.08	0.03	0.08^‡‡^	0.03	0.09	0.03	0.09‡	0.03	0.09	0.03	0.08	0.03	0.346	0.707	0.000
Defensive rebounds	0.30	0.04	0.33*	0.04	0.30^‡‡‡^	0.04	0.34*	0.04	0.29^‡‡^	0.04	0.33*	0.04	0.373	0.689	0.000
Assists	0.20	0.04	0.23*	0.04	0.21*	0.04	0.23*^,‡‡‡^	0.04	0.20	0.04	0.23*^,‡‡^	0.04	0.107	0.899	0.000
Steals	0.06	0.02	0.07*	0.02	0.06	0.02	0.07*	0.02	0.06	0.02	0.07*	0.02	0.295	0.744	0.000
Turnovers	0.13	0.03	0.12*	0.03	0.12	0.03	0.13	0.03	0.13	0.03	0.12	0.03	3.619	0.027	0.004
Blocks	0.03	0.01	0.04^‡‡,‡‡‡^	0.01	0.04	0.02	0.05*^,‡^	0.02	0.04	0.02	0.04*^,‡^	0.02	1.446	0.236	0.002
Fouls	0.20^‡‡,‡‡‡^	0.03	0.20^‡‡,‡‡‡^	0.03	0.18^‡,‡‡‡^	0.03	0.18*^,‡,‡‡‡^	0.03	0.19^‡,‡‡^	0.04	0.18*^,‡,‡‡^	0.03	0.466	0.628	0.001
Fouls drawn	0.20^‡‡,‡‡‡^	0.03	0.20^‡‡,‡‡‡^	0.03	0.18^‡^	0.03	0.19^‡^	0.03	0.18^‡^	0.03	0.18^‡^	0.03	0.071	0.931	0.000

Note: M, mean; SD, standard deviation; F, Fisher–Snedecor test; η^2^, partial eta-squared.

*Differences between win/lost groups (*p* < 0.05).

^‡^Difference with neutral court (*p* < 0.05).

^‡‡^Difference with home court (*p* < 0.05).

^‡‡‡^Difference with guest court (*p* < 0.05).

Table 3 shows the performance differences of pre-shot combinations (per possession) between winning and losing teams. Winning teams had better results in catch-and-shoot made, catch-and-shoot percentage, isolations made, and drive percentage. Moreover, neutral-court winners had better results in post-up percentage, cuts made, and cuts attempted and worse results in drives attempted. Home-court winners had better results in transitions made, transition percentage, isolation percentage, drive percentage, cuts attempted, cuts made, and cut percentage and less hand-off attempts. Away-court winners had better results in transitions attempted, transitions made, transition percentage, isolation percentage, hand-off percentage, cuts made, and cut percentage and less drive attempts. The effect size for all performance indicators was small.

**TABLE 3:** Results of pre-shot combinations (per possession).

	Neutral				Home				Guest						
	Lost		Win		Lost		Win		Lost		Win		Game location and result		
	M	SD	M	SD	M	SD	M	SD	M	SD	M	SD	F	p	η^2^
Transitions missed	0.04^‡‡,‡‡‡^	0.02	0.04^‡‡^	0.02	0.04^‡^	0.02	0.04^‡^	0.02	0.04^‡^	0.02	0.04	0.02	1.926	0.146	0.002
Transitions made	0.03^‡‡^	0.02	0.04^‡‡,‡‡‡^	0.02	0.04*,^‡^	0.02	0.05*^,‡^	0.02	0.04	0.02	0.04*^,‡^	0.02	0.371	0.690	0.000
Transition %	53.4	21.8	53.4^‡‡^	17.6	53.0	19.8	57.6*^,‡^	16.7	52.6	18.6	56.9*	19.5	2.070	0.126	0.002
Catch-and-shoot missed	0.10	0.04	0.10	0.03	0.11	0.03	0.10	0.03	0.11	0.04	0.10	0.03	0.816	0.442	0.001
Catch-and-shoot made	0.06	0.02	0.07*^,‡‡^	0.02	0.05	0.02	0.06*^,‡^	0.02	0.05	0.02	0.06*	0.02	0.268	0.765	0.000
Catch-and-shoot %	36.6	10.9	41.7*	11.7	34.4	113	40.2*	11.5	34.8	11.6	40.3*	11.9	0.135	0.874	0.000
Catch-and-drive missed	0.04^‡‡‡^	0.02	0.03	0.02	0.03	0.02	0.03	0.02	0.03^‡^	0.02	0.03	0.02	0.716	0.489	0.001
Catch-and-drive made	0.02	0.01	0.03^‡‡‡^	0.01	0.02	0.01	0.02	0.01	0.02	0.01	0.02^‡^	0.01	0.470	0.625	0.001
Catch-and-drive %	46.6	22.5	50.4	24.3	46.0	22.6	48.6	23.9	46.3	23.3	46.6	25.1	0.775	0.461	0.001
Post-up missed	0.02	0.02	0.02	0.02	0.02	0.02	0.02	0.02	0.02	0.02	0.02	0.02	0.535	0.586	0.001
Post-up made	0.01	0.01	0.02	0.01	0.01	0.01	0.02	0.01	0.01	0.01	0.02	0.01	0.024	0.976	0.000
Post-up %	40.8	30.6	47.7*	28.9	41.9	31.0	43.7	30.9	39.9	30.5	41.6	30.4	1.059	0.347	0.001
Isolations missed	0.05	0.03	0.05	0.03	0.05	0.04	0.05	0.04	0.05	0.04	0.05	0.04	0.433	0.648	0.000
Isolations made	0.03	0.02	0.04*	0.02	0.03	0.02	0.04*	0.03	0.03	0.02	0.03*	0.03	1.638	0.195	0.002
Isolation %	41.4^‡‡^	20.3	44.2	19.8	35.7^‡^	20.3	43.2*	20.1	38.3	20.0	41.6*	19.3	2.551	0.078	0.003
Hand-off missed	0.03	0.02	0.03	0.02	0.03*	0.02	0.02*	0.03	0.03	0.02	0.02	0.02	0.169	0.844	0.000
Hand-off made	0.02	0.01	0.01	0.01	0.02	0.01	0.02	0.01	0.01	0.01	0.02	0.01	0.810	0.445	0.001
Hand-off %	38.2	26.1	40.9	27.1	40.4	27.6	43.0	29.2	38.3	25.9	42.7*	29.5	0.199	0.819	0.000
Cuts missed	0.01^‡‡,‡‡‡^	0.01	0.02*^,‡‡^	0.01	0.02^‡^	0.01	0.02*^,‡,‡‡‡^	0.01	0.02^‡^	0.01	0.01^‡‡^	0.01	5.357	0.005	0.006
Cuts made	0.02	0.02	0.03*^,‡‡^	0.01	0.03	0.01	0.03*^,‡,‡‡‡^	0.02	0.03	0.02	0.03*^,‡‡^	0.02	2.282	0.102	0.003
Cut %	67.4	27.0	68.6	22.8	65.7	25.2	72.2*	22.2	64.6	26.6	70.0*	26.3	1.477	0.229	0.002
Drives missed	0.11	0.04	0.09*	0.03	0.12	0.05	0.11	0.05	0.13	0.05	0.11*	0.05	0.119	0.888	0.000
Drives made	0.09	0.03	0.10	0.03	0.09	0.03	0.10	0.04	0.10	0.04	0.10	0.04	0.780	0.458	0.001
Drive %	46.2	11.2	50.3*	11.9	44.6	11.5	48.6*	11.4	44.9	10.0	48.1*	11.7	0.299	0.741	0.000

Note: M, mean; SD, standard deviation; F, Fisher–Snedecor test; η^2^, partial eta-squared.

^‡‡^Difference with home court (p<0.05).

^‡‡‡^Difference with guest court (p<0.05).

^‡^Difference with neutral court (*p* < 0.05).

*Differences between win/lost groups (p<0.05).

Table 4 shows the performance differences for pick and defensive indicators (per possession) between winning and losing teams. Winning teams had better results in pick-and-roll-handler percentage, pick-and-roll-roller percentage, uncontested-shot percentage, contested-shot made, and contested-shot percentage. Moreover, home-court winners had more uncontested-shot made but less pick-and-roll-handler attempts and less pick-and-roll-roller attempts. Away-court teams had more uncontested-shot made but had less pick-and-roll-handler attempts and less pick-and-roll-roller attempts. The effect size for all performance indicators was small.

**TABLE 4:** Results of picks and defensive indicators (per possession).

	Neutral				Home				Guest						
	Lost		Win		Lost		Win		Lost		Win		Game location and result		
	M	SD	M	SD	M	SD	M	SD	M	SD	M	SD	F	p	η^2^
PnR handlers missed	0.08	0.04	0.06^‡‡‡^	0.03	0.08	0.04	0.07*	0.04	0.09	0.04	0.07*^,‡^	0.03	0130	0.878	0.000
PnR handlers made	0.04	0.02	0.05	0.03	0.05	0.02	0.05	0.03	0.05	0.03	0.05	0.03	0.302	0.739	0.000
PnR handler %	37.3	15.7	45.4*	15.5	40.2	13.5	44.7*	15.4	39.7	14.1	45.1*	14.6	1.886	0.152	0.002
PnR rollers missed	0.09^‡‡,‡‡‡^	0.04	0.07^‡‡,‡‡‡^	0.04	0.09^‡^	0.04	0.08*^,‡^	0.04	0.09‡	0.04	0.08*^,‡^	0.04	0.094	0.911	0.000
PnR rollers made	0.06^‡‡,‡‡‡^	0.02	0.06^‡‡‡^	0.03	0.07^‡^	0.03	0.07	0.03	0.07^‡^	0.03	0.07^‡^	0.03	0.156	0.856	0.000
PnR roller %	41.5	14.8	49.0*	15.4	43.8	13.0	48.5*	14.1	43.2	13.1	48.9*	12.9	1.179	0.308	0.001
PnP missed	0.01	0.01	0.01^‡‡,‡‡‡^	0.01	0.01	0.01	0.01^‡^	0.01	0.01	0.01	0.01^‡^	0.01	0.157	0.854	0.000
PnP made	0.01	0.01	0.01^‡‡,‡‡‡^	0.01	0.01	0.01	0.01^‡^	0.01	0.01	0.01	0.01^‡^	0.01	1.218	0.296	0.001
PnP %	34.8	34.5	38.0	35.7	31.8	34.9	32.9	36.5	30.7	35.2	34.0	37.8	0.182	0.834	0.000
Uncontested FG missed	0.07^‡‡,‡‡‡^	0.05	0.07^‡‡,‡‡‡^	0.05	0.10^‡^	0.07	0.09^‡^	0.07	0.10^‡^	0.08	0.08^‡^	0.06	1.318	0.268	0.001
Uncontested FG made	0.04^‡‡,‡‡‡^	0.02	0.04^‡‡,‡‡‡^	0.03	0.06^‡^	0.04	0.06*^,‡^	0.04	0.06^‡^	0.04	0.06*^,‡^	0.04	0.369	0.691	0.000
Uncontested FG %	36.4	16.5	42.4*	17.1	37.1*	17.0	42.4*	16.7	35.7	15.0	43.6*	16.7	1.144	0.319	0.001
Contested FG missed	0.38^‡‡‡^	0.06	0.35^‡‡,‡‡‡^	0.06	0.36	0.08	0.32^‡^	0.08	0.36^‡^	0.08	0.34^‡^	0.07	2.108	0.122	0.002
Contested FG made	0.28	0.04	0.31*^,‡‡‡^	0.04	0.27	0.05	0.30*	0.06	0.27	0.05	0.30*^,‡^	0.05	0.090	0.914	0.000
Contested FG %	43.2	5.34	47.7*	5.58	43.5	6.75	48.8*^,‡‡‡^	6.20	43.6	5.63	47.4*^,‡‡^	6.39	2.462	0.086	0.003

Note: M, mean; SD, standard deviation; F, Fisher–Snedecor test; η^2^, partial eta-squared; PnR, pick-and-roll; PnP, pick-and-pop.

^‡‡‡^Difference with guest court (*p* < 0.05).

*Differences between win/lost groups (*p* < 0.05).

^‡^Difference with neutral court (*p* < 0.05).

^‡‡^Difference with home court (*p* < 0.05).

Table 5 shows the discriminant analysis. The discriminant function obtained was statistically significant (*p* < 0.001), and it could correctly classify 84.0% of the cases. The results allowed the classification among winners and losers through: points (SC = 0.525), field-goal percentage (SC = 0.492), field goals made (SC = 0.403), defensive rebounds (SC = 402), field goals missed (SC = −0.395), three-point percentage (SC = 0.371), and rebounds (SC = 0.314).

**TABLE 5:** Discriminant function structure coefficients (SCs) and tests of statistical significance.

Game statistic	SC	Game statistic	SC
Points	0.525*	Transitions made	0.129
FG %	0.492*	FT made	0.126
FG made	0.403*	Fouls made	−0.120
Defensive rebounds	0.402*	Hand-off missed	−0.110
FG missed	−0.395*	Uncontested FG made	0.104
Three-PT FG %	0.371*	Catch-and-drive missed	−0.102
Rebounds	0.314*	FT %	0.087
Two-PT FG %	0.291	Uncontested FG missed	−0.074
Assists	0.269	PnR rollers made	0.071
Three-PT FG missed	−0.243	PnR handlers made	0.055
Three-PT FG made	0.238	Fouls drawn	0.054
Contested FG made	0.234	PnP missed	−0.042
Contested FG missed	−0.226	Post-up made	0.041
Two-PT FG missed	−0.205	Drives made	0.027
Two-PT FG made	0.194	PnP made	0.025
PnR handlers missed	−0.190	Turnovers	−0.024
PnR rollers missed	−0.189	Transitions missed	−0.018
Blocks	0.173	Catch-and-drive	−0.015
Steals	0.172	Offensive rebounds	−0.014
Drives missed	−0.163	FT missed	0.005
Catch-and-shoot missed	−0.155	Cuts missed	−0.004
Catch-and-shoot made	0.151	Hand-offs made	0.001
Cuts made	0.145	Isolations missed	0.001
Isolations made	0.129	Post-up missed	0.000

Note: FG, field goal; two-PT, two-point; three-PT, three-point; FT, free throw; PnR, pick-and-roll; PnP, pick-and-pop.

*SC, discriminant value > |0.30|.

Table 6 shows the results of the binary logistic regression analysis. The model correctly classified 79.1% of cases for home-court teams, 75.5% cases for away-court teams, and 50.3% for neutral-court teams. The main results showed that defensive rebounds (*p* = 0.043; OR = 8820.506), rebounds (*p* < 0.001; OR = 16772297.13), and three-point field-goal percentage (*p* < 0.001; OR = 1.054) were statistically significantly associated to winning for home-court teams. Defensive rebounds (*p* = 0.036; OR = 4448.926), rebounds (*p* < 0.001 OR = 9986637.185), and three-point field-goal percentage (*p* < 0.001; OR = 1.069) were statistically significantly associated to winning for away-court teams. Defensive rebounds (*p* = 0.002; OR = 7300219.697) and three-point field-goal percentage (*p* = 0.014; OR = 1.047) were statistically significantly associated to winning for neutral-court teams.

**TABLE 6:** Binary logistic regression values.

Variable	B	P	Exp (B)	95% CI Exp (B)	
				Lower	Upper
Game location: neutral					
FG %	0.176	0.623	1.192	0.591	2.404
FG made	1.241	0.959	3.458	0.000	1.768E21
Defensive rebounds	15.803	0.002	7300219.697	329.128	1.619E11
FG missed	1.345	0.949	3.839	0.000	2.139E18
Three-PT FG %	0.046	0.014	1.047	1.009	1.085
Rebounds	3.642	0.415	38.169	0.006	241970.113
Constant	−17.271	0.301	0.000		
Game location: home					
FG %	−0.126	0.678	0.882	0.487	1.596
FG made	18.990	0.346	1.767E8	0.000	2.604E25
Defensive rebounds	9.085	0.043	8820.506	1.316	59103503.63
FG missed	−25.482	0.143	0.000	0.000	5559.991
Three-PT FG %	0.052	0.000	1.054	1.025	1.083
Rebounds	16.635	0.000	16772297.13	10419.387	2.700E10
Constant	−1.307	0.926	0.271		
Game location: away					
FG %	0.060	0.829	1.062	0.614	1.838
FG made	7.360	0.695	1571.634	0.000	1.495E19
Defensive rebounds	8.400	0.036	4448.926	1.766	1.121E7
FG missed	−11.139	0.480	0.000	0.000	3.871E8
Three-PT FG %	0.067	0.000	1.069	1.040	1.099
Rebounds	16.117	0.000	9986637.185	9168.272	1.088E10
Constant	−12.081	0.347	0.000		

Note: FG, field goals; three-PT, three-point.

## Discussion

This study aimed to analyze the team’s performance differences (game-related statistics, pre-shooting combinations, and pick-and-roll performance) between winning and losing teams in different game locations (neutral court, home court, and away court) in the NBA during the COVID-19 season. We hypothesized that winners should have better shoot percentages and rebounds; more transitions and catch-and-shot success; and more pick-and-roll and uncontested shots. The results from the discriminant analysis and the binomial logistic regression confirmed the importance of some key performance indicators differentiating winning and losing teams (rebounds, defensive rebounds, field goals, or three-points). Then, the first hypothesis was accepted since the winners grabbed more rebounds and defensive rebounds and had a greater field-goal and three-point shot percentage.

In particular, a better shooting percentage in winners is similar to previous research which identified shooting percentage as one of the main discriminant variables for winners (Gómez et al., 2008a; García et al., 2014; Gómez et al., 2017; Harris & Roebber, 2019) and also supports the idea of traditional and recent studies which did not find a consistent relationship between the success of a team and the shooting percentage in two-pointers (Varca, 1980; Stavropoulos et al., 2021). However, it was not the two-point field-goal percentage but the three-point field-goal percentage the one which differentiated both teams, what reveals the growing importance of three-pointers in modern basketball (Stavropoulos et al., 2021). Regarding to shooting, it is also important to notice the higher number of free-throw attempts in winners (but not a better free-throw percentage or free-throw hits) which is consistent with the traditional idea of the importance to consistently draw fouls that lead to free-throw opportunities (Oliver, 2004; Mandić et al., 2019; Stavropoulos et al., 2021). However, the percentage seems to be only significant in away-court teams, probably due to balance a more functional aggressive behavior in home-court teams. A better result in rebounds and defensive rebounds is consistent to previous research which highlighted the importance of rebounding opportunities (Evangelos et al., 2005; Conte et al., 2017; Zhang et al., 2019; Stavropoulos et al., 2021), especially defensive rebounding to recover the possession of the ball to begin a fast-break (Gómez et al., 2008a; Gómez et al., 2008b; Sampaio & Janeira, 2003) while avoiding the other team to grab an offensive rebound with great odds of an easy or open shot (Csátaljay et al., 2017). Other game-related performance indicators that seemed to be associated to more winning opportunities, but that has not been always consistently connected, are assists and steals (Sampaio & Janeira, 2003; Gómez et al., 2008a; Gómez et al., 2017; Madarame, 2017; Çene, 2018). In our study, winners had better numbers in both classifications, what suggest a tendency toward the importance of a functional aggressive defense and a collective way of the play.

If we consider the pre-shot combinations, the second hypothesis was partially fulfilled, since the winners had better percentage and number of catch-and-shot field goals, but only there were better numbers of transitions for home-court and away-court team winners. The importance of shooting percentage has been previously discussed, and this insight provides a deeper analysis in the type of shot that could be better used to enhance the odds for winning (Puente et al., 2015; Çene, 2018; Stavropoulos et al., 2021). It is also important to consider the better results in isolations made by the winners, together with a better percentage in drives. These results suggest the importance of having skilled players in one-on-one situations to clear the offense when a collective approach has been consistently stopped by the defense. The efficacy in these actions seems to be of vital importance for the success of the team. Winners also had better results in cuts made which can be interpreted as a good way to get the ball with a spatial advantage in relation to the defense to finish with a lay-up or an uncontested shot, which highlights the importance of reduced collective structures in basketball to outscore the opponents (Lamas et al., 2015).

If we analyze the pick-and-roll situations and the uncontested shots, the winners had better results in the percentage of pick-and-roll handler and roller and better percentage in uncontested shots; thus, the third hypothesis can be accepted. The importance of pick-and-roll is out of doubt nowadays and has been repeatedly reported previously (Garganta, 2009; McGarry, 2009; Gómez et al., 2015). Moreover, this study adds a new insight into the importance of a great efficiency in the pick-and-roll: it should not be regarded as a simple functional offensive unit in basketball because its inefficiency due to a good opposition could take away odds for the victory (Lamas et al., 2015). This idea is supported by the less pick-and-roll attempts home-team and away-team winners had, what proves that it is not only to play pick-and-roll but to play it efficiently what make a team a winner. As a practical approach, the coaches should quantify the success of offensive and defensive pick-and-roll strategies in game-time to improve their knowledge of the pick-and-roll efficiency. On the contrary, pick-and-pop situations were not crucial in winners, maybe due to the less strategy types to counteract this kind of play (Garganta, 2009; McGarry, 2009; Gómez et al., 2015). An uncontested shot can be regarded as a good way to finish a possession, with more odds to convert it into scored points for the team. But it was not only the number of uncontested shots but also the percentage of success what made the difference between winners and losers. It happened the same with contested shots, what supports the idea of the crucial importance of shooting percentage both at the end of a good collective offense (with an open shot) and at the end of a possession in which the defense has been good enough to contest the shot (Puente et al., 2015; Stavropoulos et al., 2021). The teams should have good shooters and good one-on-one players to consistently have good shooting percentage both in contested and uncontested situations.

The main limitation of this study was the impossibility to balance the number of games played in each group and the prevalence of playoff games in the neutral court group. To solve this, we only included teams that participated in the NBA isolation area for the study, so only the best 22 teams of the league, with real options to reach the playoffs, were included as a sample.

## Conclusion

This research studied the common performance indicators for winning teams in different game locations (neutral court, home court, and away court). Winning teams had in common better field-goal and three-point percentage, two-point field-goal, three-point field-goal made, more free-throws attempted, more rebounds and defensive rebounds, and more assists and steals. Winners also had better results in catch-and-shot percentage and catch-and-shot made, cuts made, isolations made, and drive percentage. Winning teams had more percentage of efficiency in pick-and-rolls and contested and uncontested shots. In general, winners had a better shooting percentage based on three-pointers, catch-and-shot actions, cuts, pick-and-roll efficacy, and uncontested shots based on a better collective behavior. At the same time, teams would need players with the ability to clear those possessions in which the opponents force to an isolation or a contested shot. Coaches should focus to construct a team with good shooters, skilled players in isolations, and a good game-time pick-and-roll strategy.

## Data Availability

The original contributions presented in the study are included in the article/Supplementary Material; further inquiries can be directed to the corresponding author.
