# Corrigendum: Pre-shot combinations and game-related statistics discriminating between winners and losers depending on the game location during the NBA COVID-19 season

**DOI:** 10.3389/fphys.2023.1204888

**Published:** 2023-05-02

**Authors:** Álvaro Bustamante-Sanchez, Miguel-Angel Gomez-Ruano, Vicente Javier Clemente-Suárez, Sergio L. Jiménez -Sáiz

**Affiliations:** ^1^ Faculty of Sport Sciences, Universidad Europea de Madrid, Madrid, Spain; ^2^ Facultad de Ciencias de la Actividad Física y el Deporte, Universidad Politécnica de Madrid, Madrid, Spain; ^3^ Centre for Sport Studies, Fuenlabrada, Universidad Rey Juan Carlos, Madrid, Spain

**Keywords:** basketball, pick-and-roll, ball screen, game-related statistics, pre-shot combinations

In the published article, there was an error in **Affiliation** for Miguel-Ángel Gómez-Ruano. Instead of “affiliations 1 and 2: ^1^Faculty of Sport Sciences, Universidad Europea de Madrid, Madrid, Spain; ^2^Facultad de Ciencias de la Actividad Física y del Deporte, Universidad Politécnica de Madrid, Madrid, Spain”, it should only be affiliation 2: Facultad de Ciencias de la Actividad Física y del Deporte, Universidad Politécnica de Madrid, Madrid, Spain”. There was also an error in affiliation for Sergio L. Jiménez-Sáiz Instead of “affiliations 1 and 3: ^1^Faculty of Sport Sciences, Universidad Europea de Madrid, Madrid, Spain; ^3^ Centre for Sport Studies, Fuenlabrada, Universidad Rey Juan Carlos, Madrid, Spain” it should be only affiliation 3: ^3^ Centre for Sport Studies, Fuenlabrada, Universidad Rey Juan Carlos, Madrid, Spain”.

The authors apologize for this error and state that this does not change the scientific conclusions of the article in any way. The original article has been updated.

